# Editorial: Natural products in bone health and disease: mechanisms, therapeutics, and clinical potential

**DOI:** 10.3389/fendo.2026.1972784

**Published:** 2026-09-08

**Authors:** Kok-Yong Chin, Kok-Lun Pang

**Affiliations:** 1 Department of Pharmacology, Faculty of Medicine, Universiti Kebangsaan Malaysia, Cheras, Kuala Lumpur, Malaysia; 2 Jeffrey Cheah School of Medicine and Health Sciences, Monash University Malaysia, Subang Jaya, Selangor, Malaysia

**Keywords:** bone, herbal medicine, natural products, osteoporosis, stem cells, traditional Chinese medicine, vitamin

Osteoporosis carries a significant healthcare burden worldwide. According to the Global Burden of Disease Study 2021, low bone mineral density contributed to 219,552 deaths and 7.76 million disability-adjusted life years among postmenopausal women (1). Despite the effectiveness of existing pharmacotherapeutic agents, they are not free from side effects (2). From postmenopausal and diabetic osteoporosis to osteonecrosis and osteoarthritis, chronic skeletal diseases are characterised by overlapping pathological axes of oxidative stress, persistent inflammation, and disrupted cellular turnover. This Research Topic gathers seven articles that bridge preclinical molecular pharmacology and regenerative interventions. The coverage of these articles spans across the “gut–joint–bone” axis, cellular microenvironments, endocrine regulation, and precision stem cell therapies. Collectively, these articles attempts to answer the question how promising laboratory discoveries, such as herbal compounds, vitamins, and stem cells, could be transformed into effective and safe treatments for skeletal disorders.

Traditional Chinese medicine and natural products offer unique therapeutic advantages through their multi-component, multi-target mechanisms (3). However, we must caution against assuming that such broad pleiotropy is inherently advantageous because promiscuous interactions carry risks of off-target toxicities and diluted potency. It is essential to distinguish between theoretical pleiotropic pathway mapping and demonstrated, therapeutically relevant efficacy (4). Zhou et al. provides a comprehensive review on natural products with therapeutic potential against diabetic osteoporosis. In diabetes, the accumulation of advanced glycation end products (AGEs) and reactive oxygen species triggers a “cell death network” involving apoptosis, autophagy, and ferroptosis in osteoblasts. Zhou et al. illustrate how flavonoids, terpenoids, and polysaccharides may mitigate AGEs toxicity and restore osteoblast viability by upregulating the nuclear factor erythroid 2-related factor 2 (Nrf2)/glutathione peroxidase 4 axis and downregulating pro-inflammatory nuclear factor kappa-light-chain-enhancer of activated B cells (NF-κB) cascades.

Expanding on this network pharmacology framework, Kou et al. systematically review the molecular crosstalk among the Nrf2, NF-κB, and mitogen-activated protein kinase (MAPK) pathways in general osteoporosis. They highlight how herbal monomers, such as total flavonoids of *Drynaria fortunei*, icariin, and berberine, demonstrate potential dual-target effects by activating Nrf2 to stimulate osteogenic differentiation and suppress lipid peroxidation, while suppressing NF-κB and MAPK phosphorylation to halt osteoclastogenesis.

This systemic regulation is further extended to the “gut–joint” axis by Le et al.. Investigating the therapeutic effects of Liubao tea on osteoarthritis, Le et al. combine network pharmacology with microbiomics to show that tea-derived flavonoids, such as eupatilin and tetramethoxyflavone, significantly improve subchondral bone microarchitecture and reduce cartilage erosion in mice. Crucially, these joint-protective effects are associated with a systemic remodeling of the gut microbiota and a significant downregulation of the pyrimidine metabolism pathway, a mechanism validated via faecal microbiota transplantation. This study highlights that joint and bone health are closely related to systemic metabolic and microbial homeostasis.

At the cellular level, micronutrients act as essential cofactors involved in orchestrating bone remodelling. Recently, vitamin K2 has emerged as a supportive therapy for osteoporosis (5). A systematic review and meta-analysis of randomised controlled trials by Zhang et al. demonstrates that vitamin K2 supplementation significantly increases total osteocalcin and bone-specific alkaline phosphatase levels, while markedly reducing undercarboxylated osteocalcin and the osteoclastic marker tartrate-resistant acid phosphatase. Mechanistically, vitamin K2 acts as a cofactor for γ-glutamyl carboxylase, facilitating the γ-carboxylation of osteocalcin to promote hydroxyapatite binding and mineralization, while concurrently inhibiting bone resorption via steroid and xenobiotic receptor/pregnane X receptor signaling (5).

While vitamin K2 targets osteoblast-mediated mineralization, tocotrienols (one of the vitamin E family) demonstrate protective effects on osteocytes, the master regulators of bone remodeling. Zahanordin et al. present a perspective on how tocotrienols could protect the osteocyte lacuno-canalicular network from oxidative stress. By activating the phosphoinositide 3-kinase/protein kinase B and Nrf2 pathways, on how tocotrienols could protect the osteocyte lacuno-canalicular network from oxidative stress oestrogen deficiency-induced apoptosis and modulate their secretome. This correlates with downregulation of key osteocyte-derived Wnt inhibitors, specifically sclerostin and Dickkopf-related protein 1, along with a reduction in receptor activator of nuclear factor kappa-B ligand. Consequently, the remaining osteocytes are shifted toward a pro-anabolic and anti-resorptive state, preserving bone quality and strength.

As therapeutic strategies move toward personalisation, advanced diagnostic and clinical intervention methodologies are essential. In a large-scale clinical study of over 1,300 osteoporotic individuals in northern China, Meicen et al. establish a significant correlation between thyroid hormones (TT3, FT3, TSH), peripheral thyroid sensitivity (the FT4/FT3 ratio), and vertebral fracture risk. Crucially, these endocrine markers correlate much more strongly with volumetric bone mineral density (vBMD) measured via quantitative computed tomography (QCT) than with traditional two-dimensional DXA scans. This work highlights the potential value of incorporating peripheral thyroid sensitivity and volumetric imaging into clinical risk stratification to predict and manage fractures in euthyroid aging populations.

At the other end of the intervention spectrum, regenerative medicine offers hope for severe focal bone pathologies. such as osteonecrosis of the femoral head. Wei et al. present a systematic review and network meta-analysis of eighteen clinical trials evaluating autologous stem cell transplantation combined with core decompression (CD). Their findings indicate a dose-response relationship, wherein high-dose cell therapies (exceeding 1×10^8 cells) combined with CD significantly decrease the risk of hip joint failure (conversion to total hip arthroplasty) and prevent subchondral femoral head collapse. This clinical evidence establishes a critical biological threshold of cell counts necessary to overcome local microenvironmental depletion and achieve functional tissue repair.

The articles in this Research Topic collectively bridge experimental pharmacology and patient care. Preclinical discoveries, such as the gut-joint pyrimidine axis of Liubao tea or the osteocyte secretome modulation by tocotrienols, provide the mechanistic targets. Clinical studies, such as the thyroid regulation of bone health and high-dose stem cell parameters, define the clinical metrics. To fully realize the promise of these multi-target, personalised therapeutics, future research must transition from descriptive animal studies to standardised, disease-oriented clinical trials that integrate multi-omics, 3D bone co-cultures, and sensitive biomarker assessments. By doing so, the scientific community can translate these natural, nutritional, and cellular interventions into verified, evidence-based guidelines for musculoskeletal health.
